# The Intersection of Health Literacy and Public Health: A Machine Learning-Enhanced Bibliometric Investigation

**DOI:** 10.3390/ijerph20206951

**Published:** 2023-10-20

**Authors:** Benjamin Miranda Tabak, Matheus B. Froner, Rafael Corrêa, Thiago C. Silva

**Affiliations:** 1 School of Public Policy and Government, Getulio Vargas Foundation, SGAN 602 Módulos A,B,C, Asa Norte, Brasília 70830-020, Brazil; froner2433@gmail.com (M.B.F.); rafael.correa@fgv.br (R.C.); 2 Graduate Programme of Economics, Catholic University of Brasília, Taguatinga 71966-700, Brazil; thiagochris@gmail.com

**Keywords:** health literacy, bibliometric analysis, public health, random forest, COVID-19

## Abstract

In recent decades, health literacy has garnered increasing attention alongside a variety of public health topics. This study aims to explore trends in this area through a bibliometric analysis. A Random Forest Model was utilized to identify keywords and other metadata that predict average citations in the field. To supplement this machine learning analysis, we have also implemented a bibliometric review of the corpus. Our findings reveal significant positive coefficients for the keywords “COVID-19” and “Male”, underscoring the influence of the pandemic and potential gender-related factors in the literature. On the other hand, the keyword “Female” showed a negative coefficient, hinting at possible disparities that warrant further investigation. Additionally, evolving themes such as COVID-19, mental health, and social media were discovered. A significant change was observed in the main publishing journals, while the major contributing authors remained the same. The results hint at the influence of the COVID-19 pandemic and a significant association between gender-related keywords on citation likelihood, as well as changing publication strategies, despite the fact that the main researchers remain those who have been studying health literacy since its creation.

## 1. Introduction

Health literacy has had many meanings throughout time, with different emphases and varying levels of specificity [1,2]. In this article, we approach the field of health literacy connected to public health, aiming to identify key trends in the literature and assess the development of academic research in the area. We have opted to implement a bibliometric analysis for this study to show how the field has advanced through time, how authors have networked, and emerging trends.

We have chosen to pair health literacy with terms related to public health as a way to focus our analysis on the social impact of health literacy, looking at articles that articulate health literacy with its societal impacts. This was carried out through filters applied in the queries to limit the scope of the corpus obtained.

However, it is important to note that since health literacy is a broad concept with different meanings [1], it can refer to different ways that an individual deals with their health and health information, with functional health literacy, interactive health literacy, and critical health literacy all measuring different aspects of the concept [3,4,5]. Functional health literacy deals with the basic understanding of information, interactive health literacy measures the capacity to extract information and interpret different forms of communication, and critical health literacy refers to the capacity to critically analyze health information and use it to increase agency over one’s health decisions.

The COVID-19 scenario exacerbated the importance of providing proper guidance and information to the population to solve public health problems. However, the relationship between health literacy and public health is relevant outside of a health crisis and infodemic scenario (see [6] for a discussion on how public policies were used to combat COVID-19, one of the main health crises in recent periods, and its effectiveness in a continental country. Public policies that target improving health literacy could have increased the effectiveness of these measures). Health literacy focuses on the capacity of individuals to access and understand health information and services [1], which makes it possible for them to make appropriate decisions regarding their health [7]. The importance of health literacy, however, is broader than the individual level, as low health literacy is associated with higher mortality [8], increased hospitalizations [9], and lower vaccination rates [10], which results in higher health care costs [11] and lower productivity [1].

Health literacy plays a crucial role in patient decision-making, treatment adherence, and communication with patients. However, it is essential to recognize the potential negative aspects of health literacy. The individual capacity to self-manage can be overemphasized when discussing health literacy, without considering social networks and different sources of structural social support [12]. This higher emphasis on the individual can also lead to problems given that overconfidence in self-management capabilities can lead to individuals not seeking professional medical attention when needed [13].

This overemphasis of individuals’ capacity to manage themselves is a link between health literacy and healthism that needs proper attention. The main idea of healthism is that those from socially privileged backgrounds are more prone to overemphasizing the self-management of their own health and turning this way of acting into a lifestyle and an identity [14,15]. The focus on the individual level removes structural aspects from the equation, focusing mainly on lifestyle choices, and can lead to individuals being blamed for their illnesses, as if they are the result of a failure in their capacity to self-manage their health [16].

One example of the link between healthism and health literacy is higher critical health literacy being associated with higher vaccination hesitancy [17,18,19], which can explain cases where people with higher economic and educational levels are more prone to vaccine hesitancy [17,20]. This can be further corroborated by the fact that healthism can be linked to the spread of false information [21] and an aversion to science and modern medicine [22,23].

We applied a bibliometric analysis technique using the Bibliometrix package in R [24,25] to determine relevant authors, journals, and topics inside the corpus of articles that we selected. These aspects are capable of delineating the effects of the pandemic and its consequences on the literature.

We also used machine learning in order to identify and select the most important keywords to predict the number of citations per year of papers in the area of public health and health literacy. This approach was also implemented in the Interbank Financial Networks literature [26] and provides relevant insight on the best practices for those aiming to publish in the area.

In this article, we first outline the data corpus under scrutiny and its scope and dimensions. We then describe the methodology we employed, machine-learning-assisted regression applied to both article keywords and metadata and the bibliometric approach presented in this article.

We open our Results section with the citation likelihood, presenting the regression results. We then transition into a bibliometric evaluation, analyzing the data by their primary sources, contributing authors, geographical regions, and overarching themes.

Moving to the Discussion, we situate our research within the larger academic landscape, comparing and contrasting our findings with the existing literature on similar subjects. Here, we also articulate the limitations inherent in our study, as well as suggest avenues for future research prompted by our observations.

We conclude by synthesizing our primary insights, and encapsulating their relevance and implications. Through this article, we shed light on the scientific research on health literacy and its association with public health, as well as show the most relevant themes and keywords in the area and stimulate further research.

## 2. Materials and Methods

### 2.1. Data

We obtained the data used in this article from the Scopus, Web of Science, and Pubmed databases. As queries, we employed “health literacy”, along with terms relating to public health, with the results restricted to academic articles in English. We opted for this query to select articles that focused on the broader impact of health literacy and its positive effects on society.

We can observe the specific queries used in each database below:

- Scopus: TITLE-ABS-KEY ALL = (“Health Literacy” AND (“Public Health” OR “Health Care Policy” OR “Health Services” OR “Health Care Quality” OR “Health Policy” OR “Health Promotion” OR “Public Health Service”)) AND (EXCLUDE (PUBYEAR,2023)) AND (LIMIT-TO ( DOCTYPE,“ar”)) AND (LIMIT-TO ( SRCTYPE,“j”)) AND (LIMIT-TO ( LANGUAGE,“English”));
- Web of Science: ALL = (“Health Literacy” AND (“Public Health” OR “Health Care Policy” OR “Health Services” OR “Health Care Quality” OR “Health Policy” OR “Health Promotion” OR “Public Health Service”)) and 2023 (Exclude—Publication Years) and Article (Document Types) and English (Languages) and Article (Document Types);
- Pubmed: Search: (“Health Literacy” AND (“Public Health” OR “Health Care Policy” OR “Health Services” OR “Health Care Quality” OR “Health Policy” OR “Health Promotion” OR “Public Health Service”)) Filters: Abstract, English, from 1992–2022.

The initial search yielded a total of 9925 unique articles: 5505 from Scopus, 5150 from Web of Science, and 7102 from PubMed. Our data range from 1992 to 2022. We focused on a 30-year time frame, from 1992 to 2022, as a preliminary analysis showed that the majority of relevant research has been published from the year 2000 onwards. Certain limitations should be noted regarding our dataset. Specifically, 5 articles lack information on their respective journals, 7 are missing author names, 184 lack affiliation details, 1817 do not include the author’s keywords, and 3042 are absent of specifications regarding the corresponding author. It is worth mentioning that we limited our search to articles written in English, potentially narrowing the scope of our data.

Given that our data are sourced from reputable academic data sources, namely Scopus, PubMed, and Web of Science, we do not believe that these limitations could be substantially mitigated by consulting alternative databases.

### 2.2. Methodology

#### 2.2.1. Citation Likelihood

We used the Random Forest machine learning algorithm to establish the most relevant keywords to predict citations. The algorithm is an ensemble of different decision or regression trees, depending on the target variable. For each tree, a bootstrap sample of the data is drawn, which means selecting data points randomly with replacements from the original dataset. This bootstrapping process introduces variability among the trees. Additionally, at each node in a tree, a random subset of the variables is selected, which further ensures diversity in the decisions made by individual trees.

Given that in our analysis, the target variable of interest, the log of citations per year + 1, is continuous in nature, the Random Forest algorithm used regression trees as the base learners. Each terminal node (or leaf) in a regression tree represents a numeric value, which is the predicted outcome for data points that fall into that node; the mean of all predictions in the trees of the Random Forest is the final predicted value of the algorithm.

To conduct our analysis, we initially partitioned the collected dataset of papers into two distinct subsets. The testing set comprised 20% of the total papers, while the training set contained the remaining 80%. This stratification served as the foundation for all subsequent analytical procedures. After this division, we preprocessed the data through a variable filtering approach in order to reduce the complexity of the training data and reduce the chance of overfitting. Variables with near-zero variance, which offer limited predictive capacity, were identified and excluded. Specifically, a variable was deemed to have near-zero variance if one value was predominant, appearing in more than 98% of the observations. To address potential multi-collinearity, which can influence feature importance scores and potentially bias the selection of splits in the Random Forest ensemble, we removed predictor keywords that exhibited a Pearson correlation coefficient exceeding 0.99 with another predictor.

The selection of optimal hyperparameters was conducted through minimizing the Root Mean Squared Error (RMSE). RMSE is widely recognized for its sensitivity to large errors and its ability to provide interpretations that are directly relatable to the original scale of the data [27,28]. To fine-tune the mtry (number of attributes that each tree in the forest uses during training) parameter, we employed a repeated k-fold cross-validation strategy. In this tuning process, we used 5 k-folds, 5 separate runs, and had a tune length of 30. The forest was configured to consist of 500 trees. Through this method, an mtry value of 4 was selected, as it yielded the lowest RMSE [26]. The minimum node size used was 5, meaning that there are at least 5 observations on the terminal nodes of each regression tree, and no maximum depth value was defined; however, the final model achieved a maximum depth of 64 nodes.

We applied a white-box linear regression estimated with OLS, similar to the one employed on the Interbank Financial Networks literature [26], using the most relevant attributes identified by the Random Forest algorithm to predict the average citations per year:

(1) $$y_{i}=\alpha +\beta_{1}Age+\beta_{2}SingleAuthored_{i}+\beta_{3}QtyAuthors_{i}+\beta_{4}Keywords_{i}+\epsilon$$

where *i* refers to the paper’s ID; $y_{i}$ is the average citations per year of paper *i*; α is the intercept of the regression, the value of the dependent variable when all independent variables are set to zero; $\beta_{1}$ refers to the age of the paper; $\beta_{2}$ is a dummy variable representing whether paper *i* was written by a single author; $\beta_{3}$ is the number of authors in paper *i*; $\beta_{4}$ are dummy variables that represent whether each of the top 20 keywords for predicting average citations per year, as estimated by the Random Forest algorithm, are present in paper *i*; and ϵ represents the error term, the residuals of the regression. We use robust error clustering at the paper level and show a version with fixed effects for the age of the paper. In this way, we can make our model robust for unobserved aspects regarding individual differences amidst the papers that could impact the dependent variable, and we can also show the model taking the age variable into account and controlling for it.

#### 2.2.2. Bibliometric Analysis

This study applies bibliometric analysis to the data collected from Scopus and Web of Science on the literature on health literacy. To evaluate the state-of-the-art on the topic, we used the Bibliometrix 4.1.2 package in the statistical programming language R.

The bibliometric approach allows for a reproducible, systematic, and transparent study [24,25]. The functions of the Bibliometrix package allowed us to see the metadata trends of the vast corpus in question. This package allows for the charting of descriptive data regarding the scientific production on the chosen topic and other bibliometric methods, such as Lotka’s law.

Lotka’s law refers to a mathematical model capable of measuring the productivity of authors, assessing the contribution of different researchers to the progress of science, and evaluating the distribution of scientific production [29]. The number of authors who make n contributions in a specific field of scientific knowledge is approximately $1/n^{2}$ of those who make only one. Lotka’s law can be formally represented as follows [29]:

(2) $$x^{n}\;y=const$$

where *y* is the frequency of authors who have published *x* number of articles, *n* represents the degree of inequality in the distribution of productivity, and const represents a constant value that remains the same as *x* and *y* vary, being the total number of articles observed.

We apply this formula to quantify the distribution of scientific production in a specific field. Our main aim in applying Lotka’s law is to determine how many researchers are highly productive in the health literacy area and how many have published a low number of articles in this specific area.

## 3. Results

**Figure 1:**
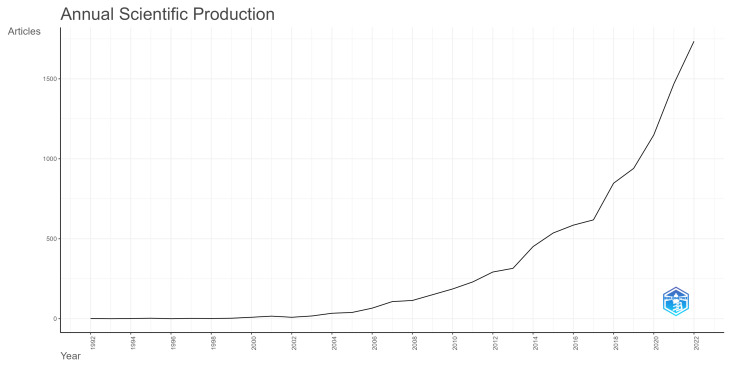
Annual scientific production.

The term health literacy gained popularity in the 1990s [30] and has been more researched as time goes on. This can also be observed in the case of studying health literacy with a focus on public health, as can be seen in Figure 1.

Figure 1 shows how the research on health literacy and public health has been expanding. This growth has been more pronounced in recent years. The mean number of articles published from 2000 to 2010 is 67.91, and from 2011 to 2022, that mean grows to 773.92. Exclusively in 2022, 1735 articles were published on the theme.

### 3.1. Citation Likelihood

**Figure 2:**
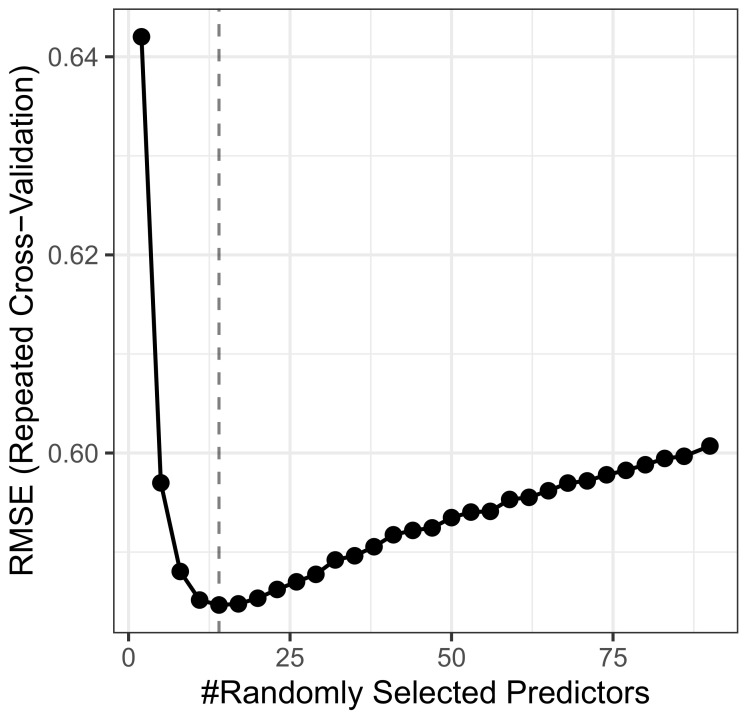
RMSE minimization value for mtry.

For the first step of selecting the hyperparameters, we defined the number of trees as 500 and then tested the resulting RMSE for each mtry, as can be seen in Figure 2, which exhibits the results of a repeated k-fold cross-validation procedure, with five k-folds being used, five independent runs, and a tune length of 30. The mtry selected was four, as it was the value that minimized the RMSE [26]. The resulting RMSE in the test set was 6.780703, a reasonable value, given that the range of the target variable is from 0 to 466.5.

The trained algorithm was then used to identify the most important keywords to predict average citations per year of each article. The top 20 keywords were then selected as the ${Keyword}_{i}$ variable for the estimation of the model of Equation (1). The resulting coefficients are presented in Table 1, with both the model presenting ${Age}_{i}$ as an independent variable on the first column and the model using fixed effects to control for the age of the paper being estimated on the second column.

In Table 1, the first coefficient in the first column shows that the age of the paper is relevant to its average citations per year, as can be expected given that established papers will be cited and, through their citations, will be read by more people, as well as the fact that seminal authors will be widely cited in the literature.

Regarding the effect of the number of authors on citations, unlike the results found in the Interbank Financial Networks literature [26], whether the article is single-authored is not a significant predictor of citations. However, the number of authors has a positive effect, indicating that collaboration among authors tends to yield positive results in the area of health literacy.

Now, concerning the coefficients for each dummy representing the presence of keywords in each article, eight of them show a positive and significant coefficient, with three of those having a *p*-value below 0.05. These keywords are Article, Cross-sectional study, Questionnaire, Male, Behavior (with a *p*-value < 0.01), COVID-19, Public health (with a *p*-value < 0.05), and Mental Health (with a *p*-value < 0.05).

It is noteworthy that the COVID-19 keyword has shown the highest coefficient in both models, despite being a relatively recent phenomenon. This makes sense because the COVID-19 pandemic and the subsequent public health crisis was an event closely related to health literacy, as the lack of information and guidelines was a big problem, especially in the first moments of the crisis. Many people were overwhelmed by accurate and inaccurate information, which was difficult to distinguish, especially given the unfamiliarity of the situation [31,32,33,34].

Other keywords with a positive coefficient that can give insight into the literature are “Questionnaire” and the “Cross-sectional study”, which indicate that this literature cites empirical studies more frequently, especially due to the fact that health literacy is often measured using questionnaires, such as the Health Literacy Questionnaire [35], the European Health Literacy Survey Questionnaire [36,37], and the Mental Health Literacy Scale [38].

Four keywords show a significant and negative coefficient. Those keywords are Human, Health knowledge attitudes practice, Female, and Surveys and questionnaires. It is noteworthy that articles containing the keyword “Male” received more citations compared with those featuring the keyword “Female”. This observed difference in citation rates within the scientific literature highlights an area that may benefit from further examination. Another relevant aspect here is the positive coefficient for the “Questionnaire” keyword and the negative coefficient for the “Surveys and questionnaires” keyword, which could indicate that the first keyword is more relevant due to it being more specific about the instrument being used, despite the fact that many surveys use questionnaires in them.

Due to inherent limitations in the capture and quantification of academic citations, the model exhibits a relatively low $R^{2}$ value. Factors that contribute to this limitation include the reputation of the authors within the scholarly community and the overall quality of the article, variables that are difficult to incorporate into a predictive model.

### 3.2. Sources

**Figure 3:**
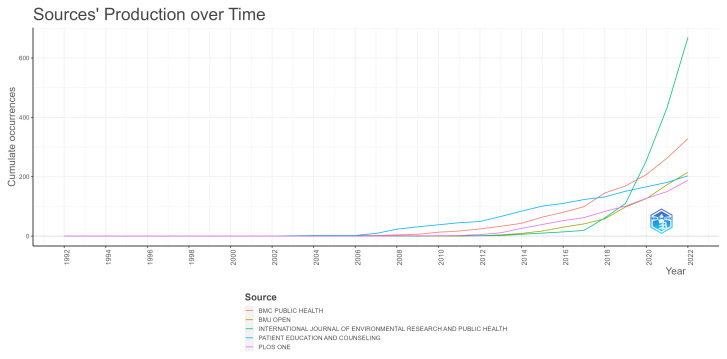
Journal dynamics throughout time.

This expansion of research on the topic also co-occurs with a significant change in the dynamics of prominent journals. In Figure 3, the sudden growth of the International Journal of Environmental Research and Public Health is evident from 2017 onward, reaching more than 500 published articles and being the most relevant source in the area by 2022. Patient Education and Counseling was the most important source from 2006 to 2017, being surpassed by BMC Public Health from 2018 to 2019, which was then surpassed by the International Journal of Environmental Research and Public Health in 2020. The International Journal of Environmental Research and Public Health presented more than 600 articles published on the topic as of 2022. We can also see the recent growth in the case of BMJ Open, which surpassed both Plos One and Patient Education and Counseling in 2022. By 2022, the most important sources were, in descending order, the International Journal of Environmental Research and Public Health, BMC Public Health, BMJ Open, Patient Education and Counseling, and Plos One, with all of them, except for Plos One, having more than 200 articles on the theme.

### 3.3. Authors

We used the functions of Bibliometrix [24,25] to identify the most prolific and most cited authors in the field. We also examined the distribution of the production of these authors over time. For this section, author disambiguation was performed manually as a way to prevent erroneous representation of authors with similar initials and surnames.

**Figure 4:**
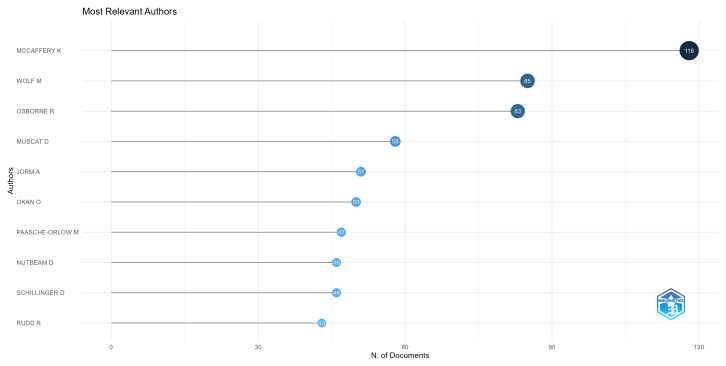
The most prolific authors.

Regarding the authors researching the topic at hand, Figure 4 shows the most prolific authors. Here, we can see the authors with the highest number of publications in our corpus. All of these authors’ publications range from 118 to 39 articles.

The top five are Kirsten McCaffery, Michael Wolf, Richard Osborne, Danielle Muscat, and Anthony Jorm. Kirsten McCaffery is a prolific author discussing themes such as over diagnosis and patient empowerment [39,40,41,42,43]. Michael Wolf is a researcher that focuses on health literacy and its impact in treatment adherence and decision making [44,45,46,47]. Richard Osborne is a researcher known for the development of the Health Literacy Questionnaire (HLQ) [35] and is active in several other empirical articles on health literacy [48,49,50,51]. Danielle Muscat is a researcher focused on health literacy and socially disadvantaged populations [52,53,54]. Anthony Jorm is one of the precursors of the research on mental health literacy [55,56], discussing problems such as stigma [57], and being a reference in the development of instruments measuring mental health literacy, such as the Mental Health Literacy Scale [38].

**Figure 5:**
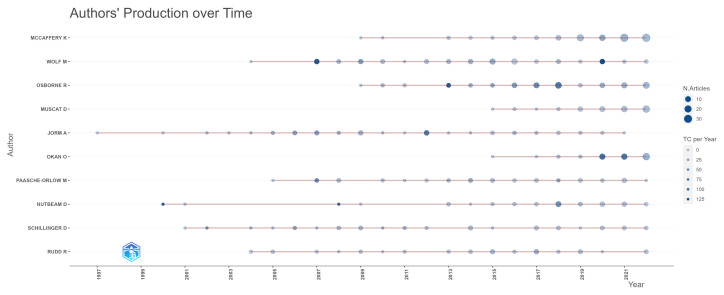
Authors’ production over time.

In Figure 5, we can see how the production of the most prolific authors is distributed over time. From this figure, it is possible to see that all the authors were still active in 2022. The authors who have been publishing in this field the longest are Anthony Jorm, who started his publications on the theme in 1997, Don Nutbeam, who had his first publication in the area by 2000, and Dean Schillinger, who published in 2001.

**Figure 6:**
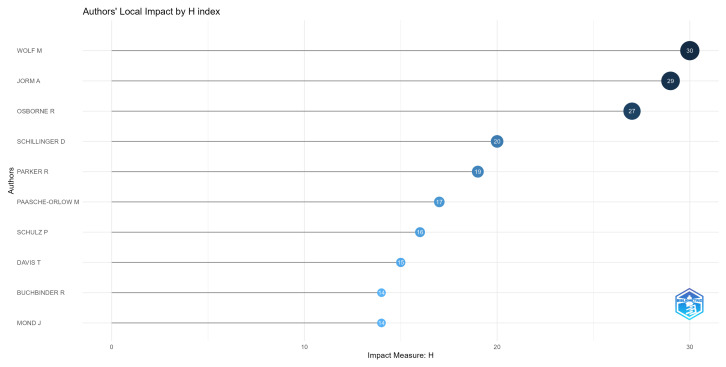
Authors with the highest H indexes.

Another essential aspect that we exhibit in Figure 6 is the authors with the highest impact, measured by their H index. Comparing Figure 5 with Figure 6, we can see that the authors with the highest h-index are also the ones that have been publishing on the theme for the longest time. The fact that Anthony Jorm, Dean Schillinger, Michael Wolf, and Richard Osborne were the four authors with the highest h-indexes and were also the ones that have been producing articles on the theme for the longest time, as shown in Figure 5, indicates that this correlation is a possible explanation.

A large proportion of the authors in Figure 4 and Figure 6 are responsible for helping to develop health literacy assessment methodologies. Richard Osborne has developed the Health Literacy Questionnaire (HLQ) to assess patient-reported outcomes related to health literacy [58]. Michael Wolf has worked on the Rapid Estimate of Adult Literacy in Medicine (REALM) [59,60]. Orkan focused on the adaptation of the European Health Literacy Survey (HLS-EU) to children [61,62,63]. Anthony Jorm has focused on different tools to evaluate mental health literacy and dementia [55,64,65].

**Figure 7:**
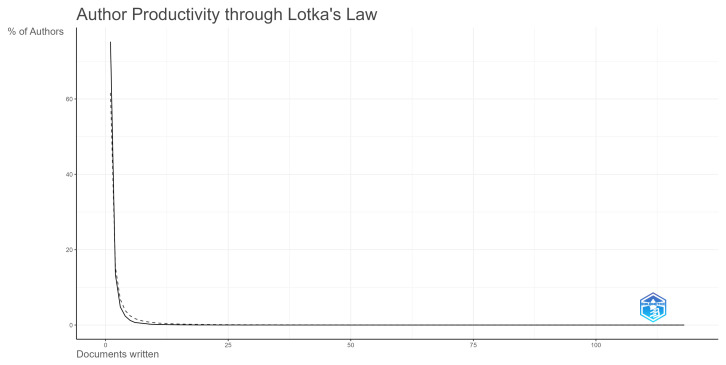
Lotka’s law.

We applied Lotka’s law to evaluate how the number of publications on health literacy is divided among authors. The graph illustrating the curve of Lotka’s law can be seen in Figure 7. The results show that 75.2% of authors have published just one article on the theme, 13.4% have published two articles, and 11.4% have published three or more articles. This shows the few prolific authors on the topic of health literacy, with a majority of authors having few contributions.

### 3.4. Region

Another important aspect of the literature is the countries in which the topic is being researched. Here, we mapped the countries according to the corresponding author of each paper. It is important to note that our corpus consists only of English articles, which reduces the number of articles from countries where English is not the native language.

**Figure 8:**
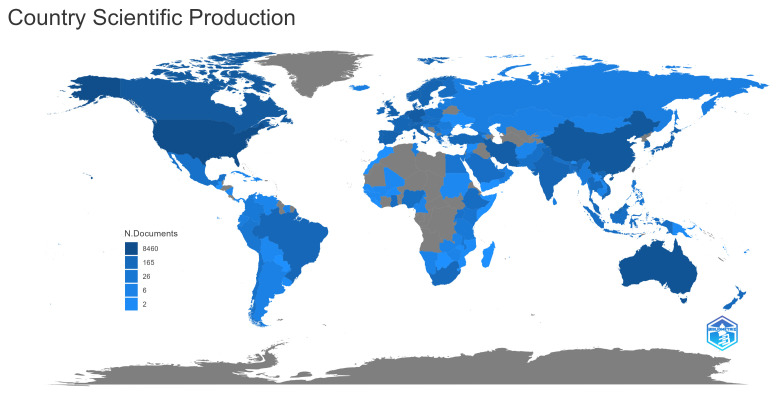
Countries’ scientific production.

Figure 8 shows a map in which each country with articles is shaded blue, the intensity of the color represents the number of articles published by corresponding authors in that country. The countries with the most articles are the USA, China, and Australia. In Europe, the countries with the most publications are the United Kingdom and Germany; in South America, Brazil has the most articles, and in Africa, the most prolific country is South Africa.

**Figure 9:**
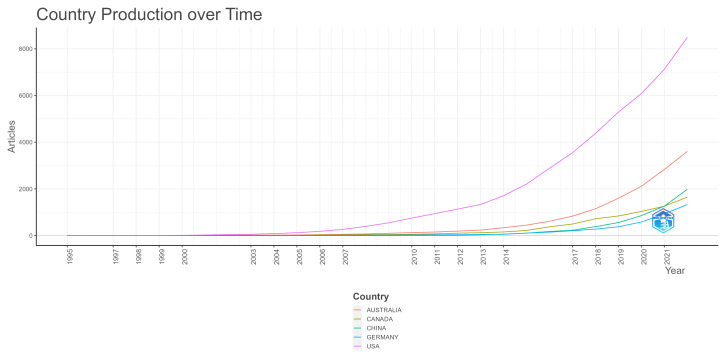
Countries with the highest number of publications.

In Figure 9, the five countries with the highest number of publications, the USA, Australia, China, Canada, and Germany, are shown with their number of articles per year. The USA is an outlier with more than 8400 articles published in 2022. Australia shows over 3500 articles in 2022, while China, Germany, and the United Kingdom have under 1900 each. This shows how the production of scientific articles on the theme under investigation is concentrated in a few specific prolific countries. Another aspect that can be seen is the recent growth of China, which became the third country with the most articles in 2021.

### 3.5. Topics

**Figure 10:**
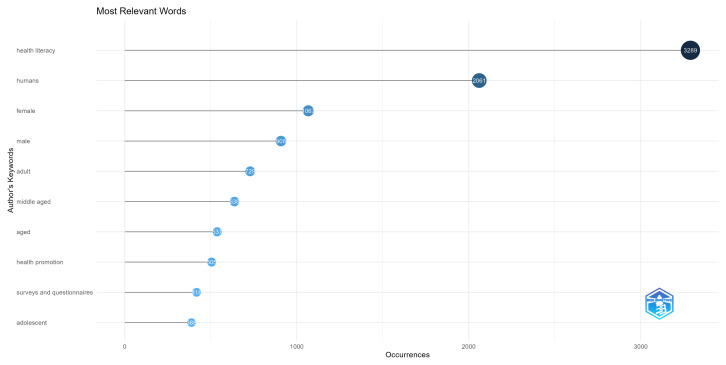
The most frequent keywords.

In order to evaluate the topics being studied by the authors, we opted to analyze the author’s keywords. We present the most frequent keywords in Figure 10. Here, we can see the terms most related to the groups being studied, such as Male or Female, Adult, Adolescent, Middle Aged, and Aged. We can also see surveys and questionnaires, as these are the most common health literacy measurement tools.

**Figure 11:**
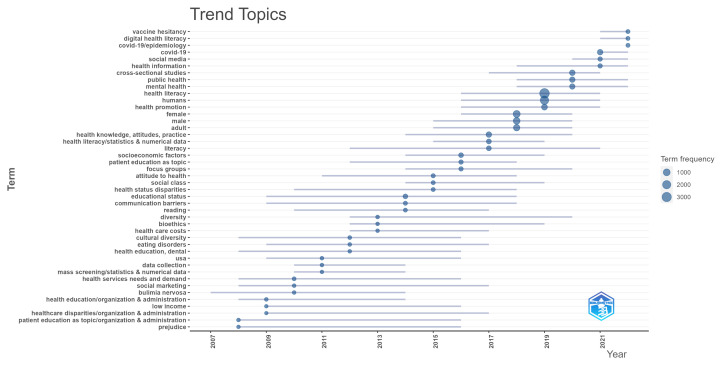
Trending keywords.

Figure 11 shows the time frame in which each topic has been primarily addressed. Each horizontal line represents the interquartile range of years, spanning from the 1st to the 3rd quartiles, during which each term was the most frequently cited or discussed. The bubble along each line is positioned at the median year, offering a more specific point within that range to indicate the central tendency of the term’s appearances over time. Here, it is possible to visualize recent topics, such as COVID-19, vaccine hesitancy, social media, digital health literacy, and mental health literacy. It is also possible to notice how some topics have started receiving less attention, such as those related to the organization and administration of health facilities.

COVID-19 and vaccination hesitancy are topics that have recently gained attention due to the pandemic and infodemic situation that increased vaccination hesitancy around the world [66]. Other topics related to the pandemic and co-occurring infodemic are the ones of social media [33,67] and mental health [68,69,70].

We also opted to visualize the most frequent words used in the articles’ titles, analyzing them isolated from other adjacent terms (as unigrams). This is another way to look into the most studied topics that might not have been indexed as keywords.

**Figure 12:**
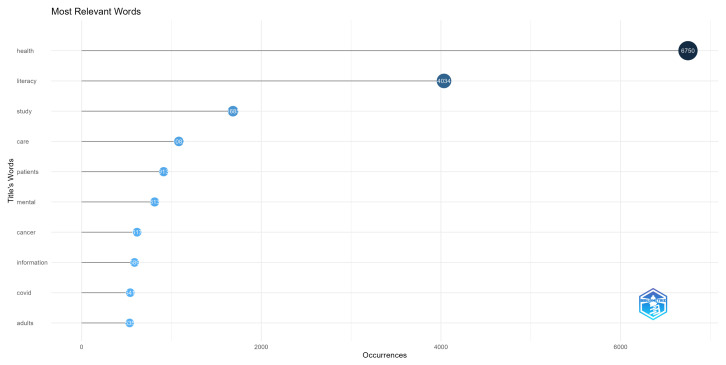
The most frequent title terms.

Figure 12 shows us some specific terms that indicate aspects being studied by health literacy researchers. COVID-19 and mental health are shown, as well as in Figure 11, indicating that while being more recent topics of study, they already appear in a substantial number of articles. Cancer also appears as a theme present in many article titles, which is consistent with research showing that health literacy is important to promote cancer detection [71] and adequate patient decision making and treatment understanding, which can lead to better results [72].

## 4. Discussion

This research used the bibliometric analysis methods present in Bibliometrix to examine the emerging trends and patterns in scientific production regarding health literacy. Our analysis sets itself apart due to its use of the Random Forest algorithm to estimate the impact of keywords on average citations per year and the focus on public health.

Other studies using bibliometric methods on health literacy have been conducted but with different focuses, such as academic production on the theme of health education and health literacy [73], or with a regional emphasis, focusing on the studies performed on the theme in Europe [74] that also find the predominance of the USA on the theme by 2008 and decides to focus on the specificity of Europe. There is also a systematic review of health literacy measurement instruments coupled with bibliometric techniques [75].

We identified that health literacy as a field has grown with exceptional intensity in the last few years. The themes of COVID-19, mental health, and social media are relevant to this expansion. Another evident aspect is the prominence of the International Journal of Environmental Research and Public Health in recent years, similar to what was found in the case of health literacy and health education [73].

These recent changes in the field, coupled with the fact that all of the most prolific authors are still active, show that this is a growing theme that is expanding and changing focus as time progresses. We also found that when looking at the authors with the highest H indexes, several of them have developed or adapted tools and methodologies for evaluating health literacy, which were then incorporated into other research.

Using the Random Forest algorithm, we showed, through the use of the OLS regression exhibited in Equation (1), that, as expected, the age of an article is relevant for its average citations per year. The number of authors is also relevant, with a positive relation to the amount of citations. Another aspect that we showed is that COVID-19 has a significant impact on citations, despite being a recent topic of study. Our model also revealed a positive coefficient associated with the keyword “Male” and a negative coefficient for the keyword “Female” in terms of citation likelihood. While these coefficients suggest an association, it is important to note that this does not establish causality, as other factors might be influencing this aspect. We also found a positive coefficient for the “Questionnaire” and the “Cross-sectional study” keywords, which indicates an interest in empirical studies.

Our analysis yields several intriguing findings that warrant further exploration. Firstly, the gender disparity in citation likelihood, evidenced by differing coefficients for the keywords “Male” and “Female”, invites scrutiny. One possible explanation could be the limited volume of articles that address gender aspects in health literacy. Further research is essential to explore these dynamics more fully and to better understand whether they reflect broader issues related to gender representation in the literature.

Secondly, the positive relationship between the number of authors and citation rates merits attention. This suggests that collaborative efforts are not merely additive but synergistic, enhancing the academic impact of a paper. It underscores the value of interdisciplinary work in this domain, thereby offering a compelling argument for collective research endeavors.

Lastly, our model indicates a favorable reception for empirical articles within the academic community. This trend could serve as a strategic guide for future research. Scholars may wish to focus on specific population subsets and adopt empirical methodologies to enhance the impact of their work.

Although our study offers important information, it has notable limitations. First, the model overlooks qualitative factors; for example, Figure 6 shows that authors who contribute to health literacy tools receive more citations, highlighting unmodeled variables that affect citation rates. Second, our dataset is confined to articles with indexed keywords in selected academic databases, potentially introducing selection bias. Finally, the study is limited by the specific search terms and time frame, limiting its applicability to areas of specialized literature such as digital health literacy or mental health literacy.

Further studies can be conducted using different corpora, tailoring them to each corpus to encapsulate different dimensions of health literacy, be it the type of health literacy, such as functional, interactive, or critical [3,4,5], or specific themes, such as mental health literacy or digital health literacy [38,76]. Another aspect that is kept out of our analysis is the qualitative aspects of the articles. Studies, such as systematic reviews, can be carried out in the area to evaluate the quality of articles on health literacy, evaluating aspects such as study design, methodology, sample size, and data collection methods.

More research is also warranted to fully understand the gender differences that our findings identified in the literature, as diagnosing gender gaps in healthcare and health-related research is essential to promote health equality.

## 5. Conclusions

The field of health literacy and public health has grown in number of publications in the last two decades, with the bulk of its growth occurring since 2015. However, we have also shown a change in the most studied topics. This is an ongoing change, and it remains to be seen how this field of study will grow with time if the new emerging themes and authors become the most cited ones.

For the time being, topics related to COVID-19 remain highly researched topics. In addition to pandemic-related themes, our findings reveal differing citation likelihoods associated with gender-related keywords. Specifically, articles tagged with the keyword “Male” showed a higher a positive and significant coefficient, while those tagged with “Female” showed a significant negative one. This difference invites further exploration to better understand the dynamics at play. Empirical studies with the keywords “Questionnaire” or “Cross-sectional study” have also shown a positive coefficient for average citation.

We have also found a recent change in the most relevant journals on the theme, with the International Journal of Environmental Research and Public Health increasing in number of articles published from 2018 onward, and in 2022, it was the most prolific journal in the literature. We have also seen a growth in production from China in the last decade, with China being the third most productive country behind the USA and Australia. These changes, coupled with the rising new themes, indicate that the literature is evolving, incorporating new topics with the entrance of new authors, reflecting the growth and diversification of the field.

This article provides a bibliometric view of the field of the intersection between health literacy and public health and introduces a predictive model that highlights the most relevant keywords and their impact on citation. This reflects the themes considered relevant by the literature and also offers potential guidelines for authors in the field. However, as in all fields, it remains to be seen how the observed dynamics and immense impact of the COVID-19 pandemic will persist throughout time.

## Figures and Tables

**Table 1:** Citation likelihood Regression Coefficients.

	Dependent Variable:	
	Average Citation per Year	
	(No F.E.)	(Paper Age F.E.)
Paper age	0.205 ***	
	(0.018)	(0.000)
Number of authors	0.119 ***	0.118 ***
	(0.036)	(0.036)
Single author	−0.166	−0.170
	(0.334)	(0.312)
Human	−1.464 ***	−1.419 ***
	(0.372)	(0.363)
Article	1.189 ***	1.341 ***
	(0.364)	(0.389)
Health knowledge attitudes practice	−1.187 ***	−1.236 ***
	(0.129)	(0.120)
Antivaccine	0.107	0.136
	(0.437)	(0.444)
Cross-sectional study	0.628 ***	0.583 ***
	(0.187)	(0.187)
Female	−0.386 ***	−0.452 ***
	(0.129)	(0.139)
Health literacy	−0.051	−0.127
	(0.135)	(0.148)
Questionnaire	1.424 ***	1.331 ***
	(0.416)	(0.417)
Healthcare	0.188	0.115
	(0.194)	(0.197)
Male	0.474 ***	0.377 ***
	(0.131)	(0.123)
Surveys and questionnaires	−0.623 ***	−0.611 ***
	(0.134)	(0.132)
Health promotion	0.051	0.029
	(0.181)	(0.154)
Behavior	0.460 **	0.413 **
	(0.179)	(0.181)
Adult	−0.183	−0.196
	(0.138)	(0.145)
COVID-19	1.839 ***	2.155 ***
	(0.412)	(0.410)
Public health	0.457 *	0.353
	(0.268)	(0.254)
Controlled study	−0.308	−0.336
	(0.231)	(0.233)
Mental health	0.339 *	0.353 *
	(0.205)	(0.201)
Education	−0.162	−0.119
	(0.233)	(0.222)
Aged	0.128	0.135
	(0.129)	(0.128)
Constant	0.617 **	
	(0.285)	
Fixed Effect	None	Paper Age
Clustered S.E.	Paper ID	Paper ID
Observations	8724	8724
R ${}^{2}$	0.047	0.059
Adjusted R ${}^{2}$	0.045	0.054

Note: * *p* < 0.1; ** *p* < 0.05; *** *p* < 0.01.

## Data Availability

Data presented in this study are publicly available on Scopus and Web of Science and can be made available upon request from the corresponding author.
